# Multi-Scale Feature Fusion for Coal-Rock Recognition Based on Completed Local Binary Pattern and Convolution Neural Network

**DOI:** 10.3390/e21060622

**Published:** 2019-06-25

**Authors:** Xiaoyang Liu, Wei Jing, Mingxuan Zhou, Yuxing Li

**Affiliations:** 1 School of Mechanical Electronic & Information Engineering, China University of Mining & Technology, Beijing 100083, China; 2 College of Geoscience & Surveying Engineering, China University of Mining & Technology, Beijing 100083, China; 3 Faculty of Information Technology and Equipment Engineering, Xi’an University of Technology, Xi’an 710048, China

**Keywords:** coal-rock recognition, completed local binary pattern, convolution neural network, feature fusion, deep learning, information theory

## Abstract

Automatic coal-rock recognition is one of the critical technologies for intelligent coal mining and processing. Most existing coal-rock recognition methods have some defects, such as unsatisfactory performance and low robustness. To solve these problems, and taking distinctive visual features of coal and rock into consideration, the multi-scale feature fusion coal-rock recognition (MFFCRR) model based on a multi-scale Completed Local Binary Pattern (CLBP) and a Convolution Neural Network (CNN) is proposed in this paper. Firstly, the multi-scale CLBP features are extracted from coal-rock image samples in the Texture Feature Extraction (TFE) sub-model, which represents texture information of the coal-rock image. Secondly, the high-level deep features are extracted from coal-rock image samples in the Deep Feature Extraction (DFE) sub-model, which represents macroscopic information of the coal-rock image. The texture information and macroscopic information are acquired based on information theory. Thirdly, the multi-scale feature vector is generated by fusing the multi-scale CLBP feature vector and deep feature vector. Finally, multi-scale feature vectors are input to the nearest neighbor classifier with the chi-square distance to realize coal-rock recognition. Experimental results show the coal-rock image recognition accuracy of the proposed MFFCRR model reaches 97.9167%, which increased by 2%–3% compared with state-of-the-art coal-rock recognition methods.

## 1. Introduction

Coal is a precious natural resource all over the world [1]. China has comparatively abundant coal resources; the nation is and will continue to be the largest coal consumer and producer in the foreseeable future [2,3]. Automatic coal-rock recognition is a critical technology for intelligent coal mining and processing [4], which is helpful for adaptive height adjustment of the shearer’s drum, the process control of fully mechanized top-coal caving, and fast coal-gangue separation in coal preparation plants [5]. Due to the constraints of geological conditions and coal mining technologies, traditional coal-rock recognition methods, such as gamma ray detection, infrared detection and radar detection, are difficult to apply in practice [6]. Considering that coal and rock have distinctive visual features, coal-rock recognition methods based on machine vision have attracted extensive attention from researchers.

Effective feature extraction of coal-rock images is a key step for coal-rock recognition methods which are based on machine vision. At present, there are two main ways of extracting features for coal-rock recognition. The first is based on mathematical models. In [7], the authors proposed the coal-rock recognition method based on wavelet transform, which extracts features of coal-rock images using a given wavelet basis. A coal-rock recognition method using wavelet-domain asymmetric generalized Gaussian models was proposed in [8], and these models are used to represent the texture features of coal-rock images. However, the features extracted in this way cannot always accurately represent coal-rock images. The second is to extract features from samples by learning. In [9], the authors proposed the coal-rock image recognition method based on dictionary learning, which is used to extract features from coal-rock images. Some methods based on dictionary learning can fully extract the features of coal-rock images and more accurately represent coal-rock images. However, this high-quality feature representation requires sufficient samples in the training process. If the number of training samples is too small, on the one hand, the feature distribution of coal-rock images cannot be truly reflected, so the feature representation ability of the coal-rock images is weak and cannot achieve satisfactory recognition accuracy; on the other hand, the recognition model is prone to overfitting during training, resulting in low generalization performance. A coal-rock recognition method based on locality-constrained self-taught learning was introduced in [10], where features are extracted from the auxiliary dataset of non-coal-rock images by the dictionary optimization model, and then the coal-rock image features are acquired by combining the locality-constrained linear coding. These methods, based on self-taught learning, solve the problem of coal-rock image samples used for training not being sufficient, but the optimization object is not directly related to coal-rock recognition and features unrelated to coal-rock may be extracted, which may cause a decrease in coal-rock recognition accuracy. Most existing coal-rock recognition methods have some defects, such as unsatisfactory performance, low robustness and narrow scope of application.

As a simple yet efficient operator, the local binary pattern (LBP) is proposed for rotation invariant texture classification in [11], which has achieved impressive recognition accuracy in many applications [12,13]. According to Local Difference Sign-Magnitude Transform (LDSMT), the Completed Local Binary Pattern (CLBP) descriptor is proposed in [14]. The descriptor CLBP can be directly utilized to extract the local texture features from the coal-rock images, but the recognition accuracy is not high enough. These traditional feature descriptors can only extract low-level features from the images.

In recent years, deep learning, especially using Convolutional Neural Networks (CNN) [15], has become an active research topic in computer vision and pattern recognition. CNNs have been proven to have the best performance in image processing, e.g., for image classification [16,17], face recognition [18] and target detection [19], thanks to its powerful automatically learning capabilities. Compared with traditional feature descriptors, CNNs can extract high-level features from coal-rock images.

In this paper, the Multi-scale Feature Fusion Coal-Rock Recognition (MFFCRR) model based on CLBP and CNN is proposed to extract and fuse the texture and deep features of coal-rock images for coal-rock recognition. The local texture features extracted by CLBP represent the low-level features of the coal-rock image, while the deep features extracted by CNN represent the high-level features. Firstly, the multi-scale CLBP feature vector is extracted from coal-rock image samples in the Texture Feature Extraction (TFE) sub-model. Secondly, the high-level deep feature vector is extracted from coal-rock image samples in the Deep Feature Extraction (DFE) sub-model. Thirdly, the multi-scale feature vector is generated by fusing the multi-scale CLBP feature vector and deep feature vector. Finally, multi-scale feature vectors are input to the nearest neighbor classifier with the chi-square distance to realize coal-rock recognition. The proposed MFFCRR model not only reduces the heavy workload of manual extraction features, but also solves the problems of unsatisfactory performance and low robustness. Experimental results show that the MFFCRR method has better performance than state-of-the-art coal-rock recognition methods.

The rest of this article is organized as follows. Section 2 is the overall structure of the MFFCRR model. Section 3 presents the proposed MFFCRR method based on CLBP and CNN. Section 4 shows a contrast of different methods with our method, the model performance and experimental results. Section 5 presents the conclusions and directions for future work.

## 2. Overview of the Proposed MFFCRR Model

**Figure 1:**
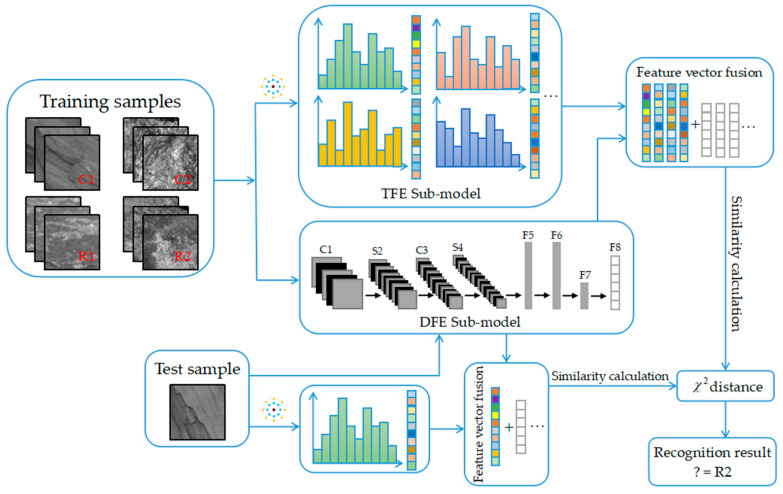
The architecture of the proposed MFFCRR (multi-scale feature fusion coal-rock recognition) model.

As shown in Figure 1, the proposed MFFCRR model is mainly composed of three parts: multi-scale feature extraction, feature fusion and recognition.

The multi-scale feature extraction part includes two paralleled steps: extracting the texture features and deep features, which are extracted in the TFE sub-model based on CLBP and the DFE sub-model based on CNN, respectively. Firstly, in the TFE sub-model, the multi-scale CLBP feature vector is extracted from coal-rock image samples, which represents texture information of the coal-rock image; Secondly, in the DFE sub-model, the high-level deep feature vector is extracted layer by layer from coal-rock image samples, which represents more abstract and macroscopic information of the coal-rock image.

After the multi-scale feature extraction is completed, the multi-scale feature vector is generated by fusing the multi-scale CLBP feature vector and deep feature vector. Finally, the multi-scale feature vectors, which are extracted from training samples and testing samples respectively, are input to the nearest neighbor classifier with the chi-square distance to realize coal-rock recognition. The following section describes these three parts of the MFFCRR model in detail.

## 3. Multi-Scale Feature Extraction, Fusion and Recognition

### 3.1. Texture Feature Extraction Sub-Model (TFE Sub-Model)

**Figure 2:**
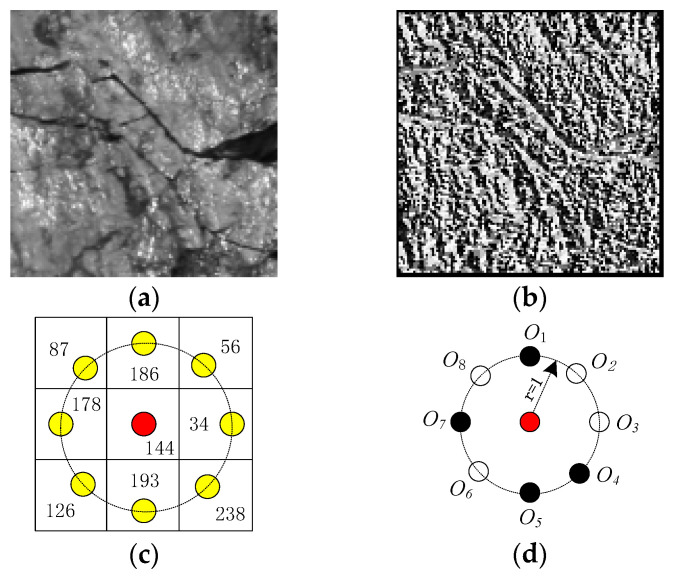
(**a**) A 128×128 anthracite gray-scale image sample; (**b**) The LBP (local binary pattern) image corresponding to this sample; (**c**) The 3×3 local structure with central pixel being 144 sampled from this sample. The red and yellow spots denote the center pixel and its 8 circularly and evenly spaced neighbors with radius 1, respectively; (**d**) The sign component of this 3×3 local structure. By thresholding, the white and black spots denote s(⋅)=0 and s(⋅)=1, respectively. It is clearly seen that the traditional LBP codes the local pattern as an 8-bit string “01001101”, starting from $O_{8}$ and coding clockwise.

Completed Local Binary Pattern (CLBP) [14] is a completed pattern of the LBP operator for texture classification based on the LDSMT, and three operators, namely CLBP-Sign (CLBP_S), CLBP-Magnitude (CLBP_M) and CLBP-Center (CLBP_C), are proposed. The CLBP_S operator is equivalent to the classical LBP. Given a pixel in the image, a traditional LBP [11] code is calculated by comparing it with its neighbors

(1) $$LBP_{P,R}=\sum_{p=0}^{P-1}s\left(g_{p}-g_{c}\right)2^{p},\;\;\;s\left(x\right)=\{\begin{array}{cc} 1, & x\geq 0\\ 0, & \;x<0 \end{array},$$

where $g_{c}$ and $g_{p}$ (p=0, … ,p−1) denote the gray values of the central pixel and its circularly symmetric neighbors respectively. R denotes the radius of the neighborhood, and P denotes the total number of the neighbors. Assuming that the coordinate of $g_{c}$ is (0,0), then the coordinates of $g_{p}$ are (Rcos(2πp/P),Rsin(2πp/P)). Note that, if the neighbors are not in the image grids, the gray values of neighbors can be estimated by interpolation. Figure 2 shows an example of LBP coding.

After LBP operator values are calculated, the histogram of LBP values is built to represent the texture features of the image. However, if the image is rotated, the LBP value will be changed; meanwhile, this will correspondingly result in the different image texture feature. Hence, we cannot guarantee the rotation invariance. In addition, the ${\mathrm{LBP}}_{P,R}$ operator (Equation (1)) produces $2^{P}$ distinct output values, which will cause the corresponding histogram to be too sparse and contain a lot of redundant information.

To decrease the redundant information of the texture features and achieve rotation invariance, the rotation invariant uniform pattern (it is the most effective pattern in some patterns introduced in [11]) and the following operator is proposed:

(2) $$LBP_{P,R}^{riu2}=\{\begin{array}{ll} \sum_{p=0}^{P-1}s\left(g_{p}-g_{c}\right), & if\;\;\;\;\;U\left(LBP_{P,R}\right)\leq 2\\ P+1, & \mathrm{otherwise} \end{array},$$

where

(3) $$U\left(LBP_{P,R}\right)=\left|s\left(g_{P-1}-g_{c}\right)-s\left(g_{0}-g_{c}\right)\right|+\sum_{p=1}^{P-1}\left|s\left(g_{p}-g_{c}\right)-s\left(g_{p-1}-g_{c}\right)\right|,$$

$U\left(LBP_{P,R}\right)$ denotes the number of spatial transitions (bitwise 0/1 changes) and superscript “riu2” denotes the rotation invariant uniform pattern with U≤2. Similarly, the histogram of $LBP_{P,R}^{riu2}$ values is built to represent the local image texture feature. The $LBP_{P,R}^{riu2}$ operator just has P+2 different output values in comparison to $LBP_{P,R}$. Hence, the dimension of the histogram and the redundant information of the texture features will be decreased. Meanwhile, we can also acquire the LBP image in the above process.

The LDSMT [14,20] is defined as:

(4) $$d_{p}=s_{p}*m_{p}\;\mathrm{and}\;\{\begin{array}{l} s_{p}=sign\left(d_{p}\right)\\ m_{p}=\left|d_{p}\right| \end{array},$$

where $d_{p}=g_{p}-g_{c}$ and $s_{p}=\{\begin{array}{ll} 1, & d_{p}\geq 0\\ -1, & d_{p}<0 \end{array}$. Apparently, $d_{p}$ is decomposed into two components: the sign and magnitude components. $m_{p}$ and $s_{p}$ denote the magnitude and sign of $d_{p}$ respectively. Namely, $m_{p}$ represents the magnitude change of gray values between the central pixel and the circularly symmetric neighbor, and $s_{p}$ represents the sign change. Obviously, the operator CLBP_S (namely LBP) only defines the sign component and does not consider the magnitude change. Consequently, CLBP [14] denotes a completed LBP.

The CLBP_S operator is the same as the traditional LBP defined in Equation (1). Namely, the $CLBP\_S_{P,R}^{riu2}$ operator also has P+2 different output values.

In order to code the operator CLBP_M in a consistent format with CLBP_S, it is defined as follows:

(5) $$CLBP\_M_{P,R}=\sum_{p=0}^{P-1}s\left(m_{p}-c\right)2^{p},$$

Here, c denotes the average value of $m_{p}$ from the entire image. Similar to $LBP_{P,R}^{riu2}$, the rotation invariant uniform pattern of the operator $CLBP\_M_{P,R}$ can also be defined, denoted by $CLBP\_M_{P,R}^{riu2}$. Meanwhile, the $CLBP\_M_{P,R}^{riu2}$ operator also has P+2 different output values.

The central pixel, which reflects the image local gray-scale, also has available information. To make the operator CLBP_C consistent with CLBP_M and CLBP_S, it is defined as:

(6) $$CLBP\_C_{P,R}=s\left(g_{c}-c_{I}\right),$$

where the threshold $c_{I}$ denotes the mean gray value of the entire image. It is clearly seen that the $CLBP\_C_{P,R}$ image is a binary image. In other words, the $CLBP\_C_{P,R}$ operator just has 2 different output values.

The three operators, namely CLBP_S, CLBP_M and CLBP_C, could be combined. Hence, a 3-D joint histogram of them can be built, denoted by “CLBP_ S/M/C”. As a very powerful tool for local texture analysis, multi-scale analysis can be utilized to improve recognition accuracy, which could combine the available information provided by multiple operators of diverse (R,P).

In this paper, the joint distribution $CLBP\_S_{P,R}^{riu2}/M_{P,R}^{riu2}/C_{P,R}$, shorthand for $CLBP_{P,R}$, is used to characterize the texture features of each coal-rock image. The multi-scale CLBP: $CLBP_{P_{1},R_{1}}+\cdots +CLBP_{P_{n},R_{n}}$, shorthand for Multi-CLBP, is used to extract the texture features from each coal-rock gray-scale image. Firstly, calculating the histograms of the $CLBP\_C_{P,R}$ and $CLBP\_S_{P,R}^{riu2}$ codes separately, a joint 2-D histogram of the $CLBP\_S_{P,R}^{riu2}/C_{P,R}$ code is acquired by concatenating the two histograms together. Then, calculating the histograms of the $CLBP\_M_{P,R}^{riu2}$ code, we concatenate the three histograms together and build a 3-D joint histogram. Finally, the 3-D joint histogram is transformed into a vector, denoted by $CLBP\_S_{P,R}^{riu2}/M_{P,R}^{riu2}/C_{P,R}$.

By applying the multi-scale CLBP, the local texture information can be captured effectively on diverse scales. In the TFE sub-model, we use the multi-scale CLBP to extract the texture features of the coal-rock image. Meanwhile, the experiment (see Section 4.3.1) also demonstrates that better recognition results can be acquired than utilizing single-scale CLBP. 

### 3.2. Deep Feature Extraction Sub-Model (DFE Sub-Model)

**Figure 3:**
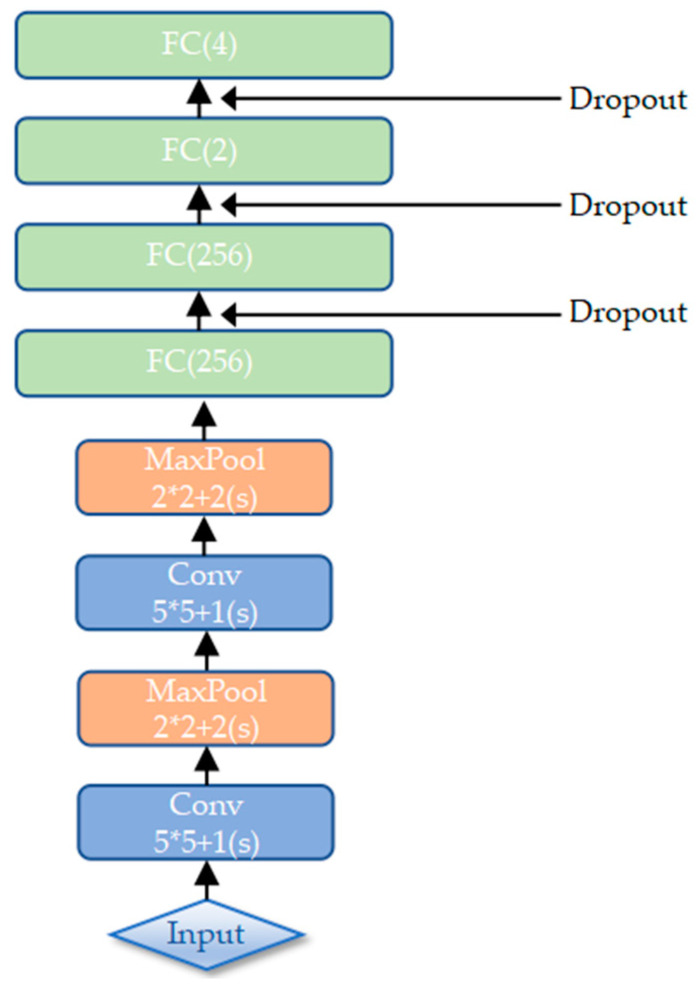
The architecture of the TFE (Texture Feature Extraction) sub-model.

After extracting the local texture features by multi-scale CLBP in the TFE sub-model, we extract the deep features from each image using CNN in the DFE sub-model. The DFE sub-model adopted in our MFFCRR model is designed based on the classic LeNet-5 network [21], whose architecture is shown in Figure 1 and Figure 3. It contains six learned layers, namely two convolutional layers (C_1_, C_3_) and four fully connected layers (F_5_, F_6_, F_7_, F_8_); spatial pooling operation is carried out by two max-pooling layers (P_2_, P_4_) which follow two convolutional layers respectively; the Parametric Rectified Linear Unit (PReLU) non-linearity is applied to the output of each convolutional and fully connected layer. In this paper, the deep features are extracted from the last fully connected layer.

Below, we describe our network’s architecture in detail and the two ways reducing overfitting.

#### 3.2.1. The Architecture of the DFE Sub-Model

The input of our network is a fixed-size 28×28 gray-scale image (in order to better adapt this network, we resize the 128×128 image to 28×28). The first layer of the sub-model is a convolution layer, which applies a convolution kernel of 5×5 and outputs 32 images of 24×24 pixels. This layer is followed by a max-pooling layer, and 2×2 sliding windows with a stride of 2 pixels are used for max-pooling to reduce the image to half of its size, namely outputting 32 images of 12×12 pixels. The second convolutional layer performs 64 convolutions with a 5×5 kernel to map the previous layer and outputs 64 images of 8×8 pixels. This layer is followed by another max-pooling layer, again with a 2×2 kernel to output 64 images of 4×4 pixels. The second max-pooling layer is followed by four fully connected layers: the first two have 256 neurons each, the third and last have 2 and 4 neurons respectively. The outputs are generated from the last fully connected layer, where the deep features are extracted.

The first convolutional layer aims to learn elementary visual features for coal-rock recognition. Further, the convolution operation is expressed as

(7) $$y^{j\left(r\right)}=\max\left(0,\sum_{i}k^{ij\left(r\right)}*x^{i\left(r\right)}+b^{j\left(r\right)}\right)+a_{i}\min\left(0,\sum_{i}k^{ij\left(r\right)}*x^{i\left(r\right)}+b^{j\left(r\right)}\right),$$

where $x^{i}$ and $y^{j}$ are the i-th input feature map and the j-th output feature map, respectively. $k^{ij}$ denotes the convolution kernel between the i-th input feature map and the j-th output feature map. ∗ represents convolution operation. $b^{j}$ denotes the bias of the j-th output feature map. Weights in the higher convolutional layer of our network are locally shared to learn different middle level visual features in different regions [22]. r in Equation (1) denotes a local region where weights are shared. We use PReLU non-linearity ($f(x)=\max\left(0,x\right)+a_{i}\min\left(0,x\right)$) as the activation function of our network, which is detailed as follows.

**Figure 4:**
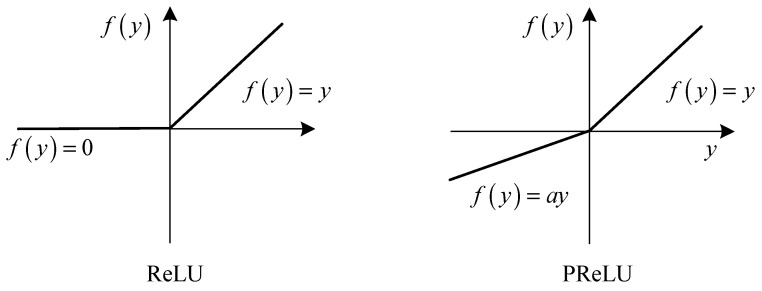
ReLU (Rectified Linear Unit) and PReLU (Parametric Rectified Linear Unit). For PReLU, the coefficient of the negative part is adaptively learned.

The PReLU improves our model fitting by adaptively learning the parameters of the rectifiers, which follows every convolutional and fully connected layer. As a new generalization of Rectified Linear Unit (ReLU), PReLU is proposed by He et al. [23] and computed as

(8) $$f\left(y_{i}\right)=\{\begin{array}{cc} y_{i}, & \mathrm{if}\;\;\;y_{i}>0\;\;\\ a_{i}y_{i}, & \mathrm{if}\;\;\;y_{i}\leq 0 \end{array},$$

where $y_{i}$ is the input of the nonlinear activation function f on the i-th channel. $a_{i}$ is a coefficient, which controls the slope of the negative part. The subscript i in $a_{i}$ indicates that the nonlinear activation can vary on different channels. If $a_{i}=0$, the activation function becomes ReLU [24]; if $a_{i}$ is a learnable parameter, it is denoted as Parametric ReLU (PReLU). The shapes of ReLU and PReLU are showed in Figure 4. In this paper, we use $a_{i}=0.25$ as the initialization (empirically chosen).

The max-pooling layer reduces the spatial resolution of the feature map outputted from the previous layer (the convolutional layer), and max-pooling is formulated as

(9) $$y_{j,k}^{i}=\underset{0\leq m,n<s}{\max}\left\{x_{j\cdot s+m,k\cdot s+n}^{i}\right\},$$

where each neuron in the i-th output feature map $y_{i}$ pools over a s×s non-overlapping local region (the pooling unit) in the i-th input feature map $x_{i}$.

Four fully connected layers are set in our network, which are used for extracting the high-level deep features of the coal-rock image. The fully connected layer takes the function

(10) $$y_{j}=\max\left(0,\sum_{i}x_{i}\cdot w_{i,j}+b_{j}\right)+a_{i}\min\left(0,\sum_{i}x_{i}\cdot w_{i,j}+b_{j}\right),$$

where x and w denote the neurons of the previous layer and weights in the current layer, respectively. Each fully connected layer is followed by the PReLU non-linearity.

The loss in our network is computed using cross entropy, which is used for constraining the coal-rock recognition task. The cross-entropy loss can be calculated as

(11) $$L\left(\theta \right)=-\frac{1}{m}\left[\sum_{i=1}^{m}\sum_{j=1}^{k}1\left\{y^{\left(i\right)}=j\right\}\log\frac{e^{\theta_{j}^{T}x^{\left(i\right)}}}{\sum_{l=1}^{k}e^{\theta_{l}^{T}x^{\left(i\right)}}}\right]+\frac{\lambda }{2}\sum_{i=1}^{k}\sum_{j=0}^{n}\theta_{ij}^{2},$$

where m and k denote the number of the labeled samples and classes, respectively. $y^{\left(i\right)}\in \left\{1,2,\cdots ,k\right\}$ corresponds to the class label of the sample $x^{\left(i\right)}\in R^{n+1}$. $\theta_{1},\theta_{2},\cdots ,\theta_{k}\in R^{n+1}$ are the parameters of the loss function. The term $\frac{2}{\lambda }\sum_{i=1}^{k}\sum_{j=0}^{n}\theta_{ij}^{2}$ is used for the weight decay.

Our network uses the Adam [25] stochastic optimization algorithm to perform parameter updates. Adam is an efficient update algorithm because information is only used for the main and secondary moments of the gradient, which is easier to perform than the back-propagation algorithm [26].

#### 3.2.2. Reducing Overfitting

Generally, the deep model needs to learn a larger number of parameters during training, which makes it more prone to overfitting. We research the following two ways in which to combat this problem.

We artificially enlarge the dataset by rotating the coal-rock image, which is one of the easiest and most common ways to reduce overfitting. The amount of data available in our dataset is not sufficient to extract the deep features of the coal-rock image; therefore, we rotate the coal-rock image from 30 degree to 330 degree with an interval of 30 degree. This method of data augmentation is applied to our network, which effectively prevents overfitting. For each image, 11 additional rotation images are generated. The coal-rock image is also flipped horizontally, which is another way of data augmentation applied to the DFE sub-model.

Additionally, we use dropout [27,28] in the first three fully connected layers, which is an efficient way of reducing overfitting. Dropout sets the output of each hidden neuron to zero with probability 0.5, and then the neurons which are “dropped out” do not conduce to the forward propagation and do not participate in backward propagation. It is clear that a different architecture is sampled by the network for each input, but these different architectures share identical weights. Hence, dropout can effectively prevent complex co-adaptations of the training data. In this paper, we use 50% dropout (empirically chosen).

### 3.3. Multi-Scale Feature Fusion and Recognition

In this paper, we designed a straightforward way to fuse the features extracted in the TFE sub-model and the DFE sub-model, namely, concatenating the feature vectors. Due to the facts that the local texture feature which has been extracted belongs to the low-level features of the coal-rock image, while the extracted deep features belong to the mid- and high-level features of the coal-rock image, when combined, the overall coal-rock recognition performance will be improved.

Firstly, in the TFE sub-model, the multi-scale CLBP feature vector (the texture feature vector) is extracted from the coal-rock image samples, denoted as the feature vector H; Secondly, in the DFE sub-model, the deep feature vector is extracted from the coal-rock image samples, denoted as the feature vector D. Then, the feature vectors H and D are normalized to $H^{*}$ and $D^{*}$, respectively; Finally, the weighting factors μ and δ are added to $H^{*}$ and $D^{*}$ respectively, and the multi-scale feature vector is generated by concatenating these two feature vectors $H^{*}$ and $D^{*}$, denoted as $X=\left(\mu H^{*},\delta D^{*}\right)$.

After generating the multi-scale feature vector X, the nearest neighbor classifier (NNC) with the chi-square distance is utilized to recognize coal-rock images. In other words, the distance between two normalized multi-scale feature vectors $X_{1}$ and $X_{2}$ was measured using the chi-square distance. Given two feature vectors $X_{1},\;X_{2}\in R^{d}$, the chi-square distance is defined as [14,20]:

(12) $$\chi^{2}\left(X_{1},X_{2}\right)=\sum_{i=1}^{d}\frac{{\left(X_{1i}-X_{2i}\right)}^{2}}{\left(X_{1i}+X_{2i}\right)},$$

where, $X_{1i}$ and $X_{2i}$ are the i-th elements of feature vectors $X_{1}$ and $X_{2}$, respectively. If $\chi^{2}\left(\cdot \right)$ is smaller, then the similarity between $X_{1}$ and $X_{2}$ is higher, which means that the probability that two coal-rock images belong to the same class is higher.

## 4. Experimental Results and Discussion

### 4.1. Dataset

**Figure 5:**
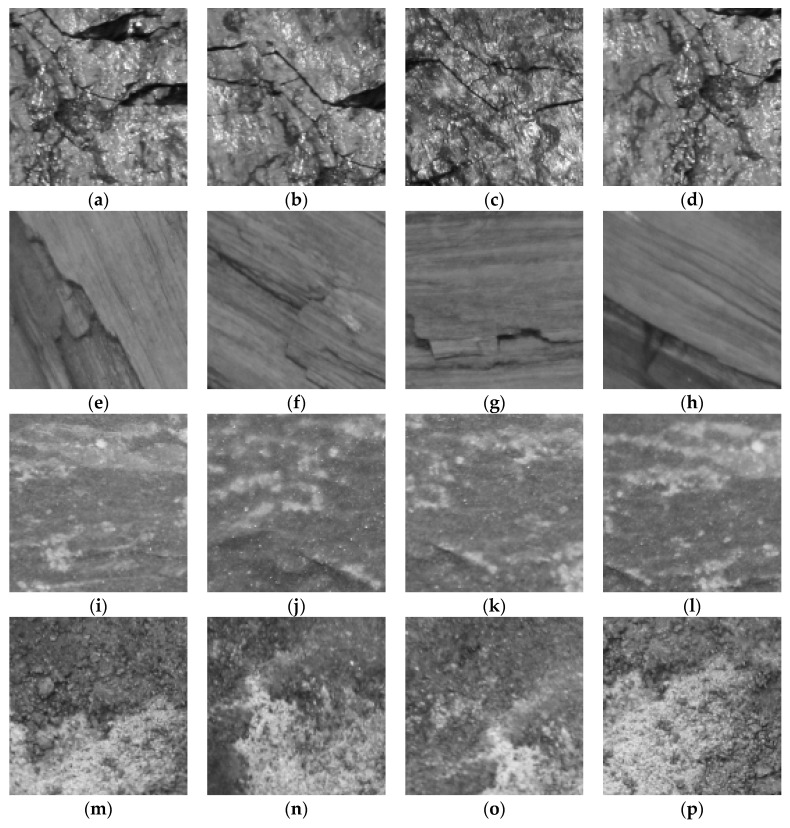
(**a**–**d**) The anthracite image samples under different illumination and viewpoints; (**e**–**h**) The lignite image samples under different illumination and viewpoints; (**i**–**l**) The mudstone image samples under different illumination and viewpoints; (**m**–**p**) The sandstone image samples under different illumination and viewpoints.

In order to evaluate performance of the proposed MFFCRR model, we implemented the experiments on an image dataset of coal-rock (CR dataset). This dataset consists of 4800 coal-rock gray-scale images of 128×128 pixels, which are collected under different illuminations and from viewpoints. There are four classes of coal-rock: lignite, anthracite, mudstone and sandstone; each has 1200 gray-scale images. Eighty percent of the samples are used for training and 20% for testing, i.e., 3840 training samples and 960 testing samples. Figure 5 shows some coal-rock examples images from four different classes (anthracite, lignite, mudstone and sandstone).

### 4.2. Evaluation Metrics

Two usual evaluation metrics, accuracy and macro-average F1, are used to accurately evaluate the MFFCRR model performance. They are computed based on the following four situations [29]:

- True Positive (TP) denotes the number of correctly recognized examples that belong to the class.
- True Negative (TN) represents the number of correctly recognized examples which do not belong to the class.
- False Positive (FP) denotes the number of incorrectly recognized examples that belong to the class.
- False Negative (FN) represents the number of incorrectly recognized examples which do not belong to the class.

Hence, the accuracy is defined as

(13) $$\mathrm{Accuracy}=\frac{TP+TN}{TP+FP+TN+FN}.$$

Sometimes, only using accuracy does not truly reflect the model performance, so the precision, recall and F1 score are introduced to comprehensively evaluate the MFFCRR model. For multi-class tasks, the performance evaluation of the proposed method should consider the prediction results of each class. Macro-average F1 represents the average of the F1 scores of all classes, which is used to efficiently evaluate the MFFCRR model performance. The precision, recall, F1 score and Macro-average F1 can be computed as follows [30]:

(14) $$\mathrm{Precision}=\frac{TP}{TP+FP},$$

(15) $$\mathrm{Recall}=\frac{TP}{TP+FN},$$

(16) $$F_{1}=\frac{2\times \mathrm{Precision}\times \mathrm{Recall}}{\mathrm{Precision}+\mathrm{Recall}},$$

(17) $$\mathrm{Macro-average}\;\;\;F_{1}=\frac{1}{k}\sum_{i=1}^{k}F_{1i},$$

where k represents the number of classes, and $F_{1i}$ denotes the F1 score of the i-th class.

### 4.3. Parameter Settings

#### 4.3.1. Parameters of the TFE Sub-Model

In this experiment, we studied the effect of the parameters P and R on the MFFCRR model. For the parameters P and R of the TFE Sub-model, we choose the three common combinations of (P, R) (namely (8,1), (16,2), and (24,3)) [14] to carry out the experiment. The three 2-scale combinations and one 3-scale combination are used for constructing the multi-scale CLBP. Table 1 shows the experimental results with macro-average F_1_ and accuracy metrics at three single-scale and four multi-scale combinations. As seen in Table 1, the MFFCRR model using the multi-scale CLBP has better performance than when single-scale CLBP is used. Further, the model based on CLBP of this 2-scale combination ((8,1) + (24,3)) gets the best recognition accuracy, 97.9167%, and a macro-average F_1_ score 97.3333%, respectively. Nevertheless, the performance of the model based on CLBP of the 3-scale combination ((8,1) + (16,2) + (24,3)) degrades a little, because more unstable distribution patterns are generated. Experimental results show the multi-scale CLBP is a powerful tool to enhance the performance of the proposed MFFCRR model.

#### 4.3.2. Parameters of the DFE Sub-Model

**Figure 6:**
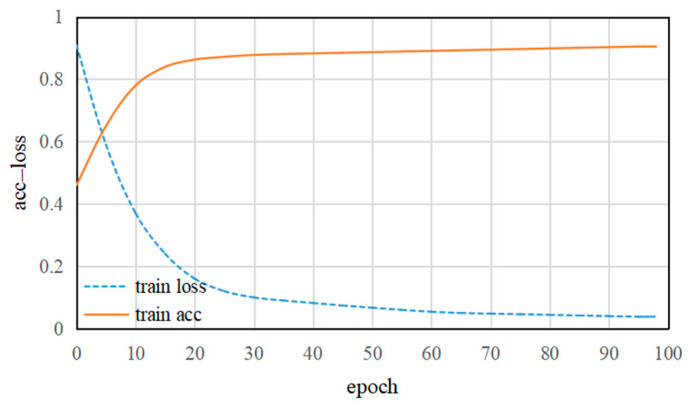
The training accuracy and training loss of the DFE (Deep Feature Extraction) network.

In order to efficiently train the DFE sub-model, the data augmentation is used throughout the whole training process (see Section 3.2.2). Figure 6 shows the training accuracy and training loss in the DFE sub-model. Clearly, with an increase in training epochs, the DFE sub-model gradually converges. As can be seen from Figure 6, with the increase of training epochs, the training loss takes about 95 epochs to reach convergence and training accuracy is close to 90% after 95 epochs. This indicates that the deep features learned by CNN can be effectively extracted from the last fully connected layer.

After performing several experiments for the recognition performance of the MFFCRR model, the hyperparameters of the DFE sub-model were obtained (summarized in Table 2). In order to reduce overfitting, we use 50% dropout (see Section 3.2.2). In addition, the DFE sub-model is trained with Adam optimizer by setting $\varepsilon =1e^{-8}$, $\beta_{1}=0.9$ and $\beta_{2}=0.999$.

#### 4.3.3. Parameters of the Multi-Scale Feature Fusion

**Figure 7:**
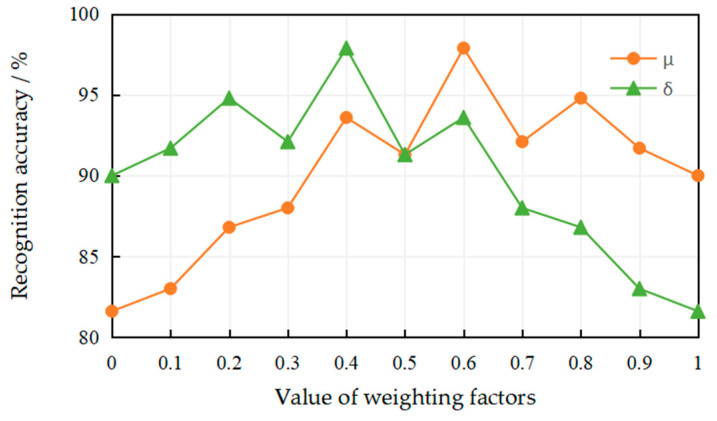
The effect of the value of the weighting factors on the recognition accuracy.

In this experiment, we studied the effect of the weighting factors μ and δ (μ,δ∈[0,1]). As two necessary parameters, the weighting factors μ and δ are used to fuse the texture feature vector and the deep feature vector (two normalized feature vectors) extracted from the coal-rock image samples, generating the multi-scale feature vector $X=\left(\mu H^{*},\delta D^{*}\right)$. Figure 7 shows the recognition accuracy at different values of the weighting factors μ and δ. As can be seen from Figure 7, using only the texture feature vector (μ=1) or the deep feature vector (δ=1) on the MFFCRR model cannot acquire better recognition accuracy. Obviously, when μ=0.6 and δ=0.4, the best recognition accuracy is acquired.

### 4.4. Implementation Details

#### 4.4.1. Activations

The activation function is necessary for state-of-the-art networks, and significantly affects the performance of the model. As one of the most common activation functions, we introduce ReLU non-linearity (f(x)=max(0,x)) to compare with PReLU non-linearity in the experiment. Table 3 shows the experimental results with macro-average F1 and accuracy metrics. As shown in Table 3, PReLU non-linearity offers better performance.

#### 4.4.2. ROC Curve

**Figure 8:**
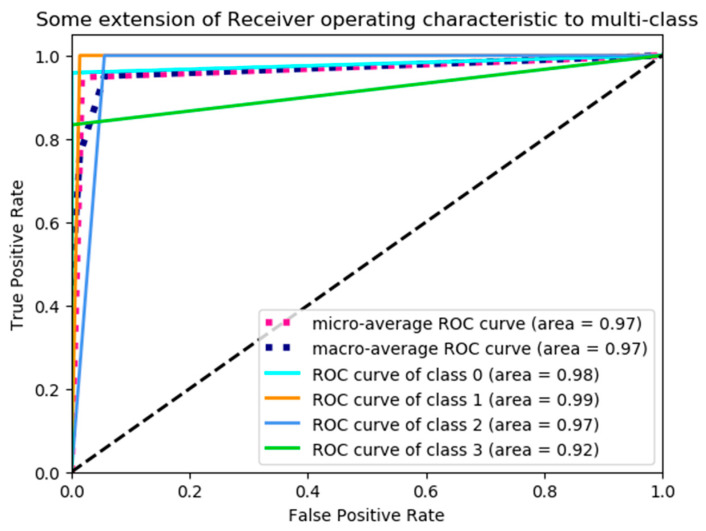
Receiver operating characteristic (ROC) Curve.

For the multi-class image recognition task, the receiver operating characteristic (ROC) curve is also an important factor to evaluate the performance of the model [30,31,32]. Hence, the ROC curves are shown in Figure 8. As seen in Figure 8, there are six curves; two of them are at an average level, and the other four are at a certain level. These two average curves show averages of areas under the curves at the macro- and micro-levels; these four curves at a certain level show the area under the curve of each class, where the class labels 0, 1, 2 and 3 correspond to lignite, anthracite, sandstone and mudstone, respectively.

#### 4.4.3. White Gaussian Noise

**Figure 9:**
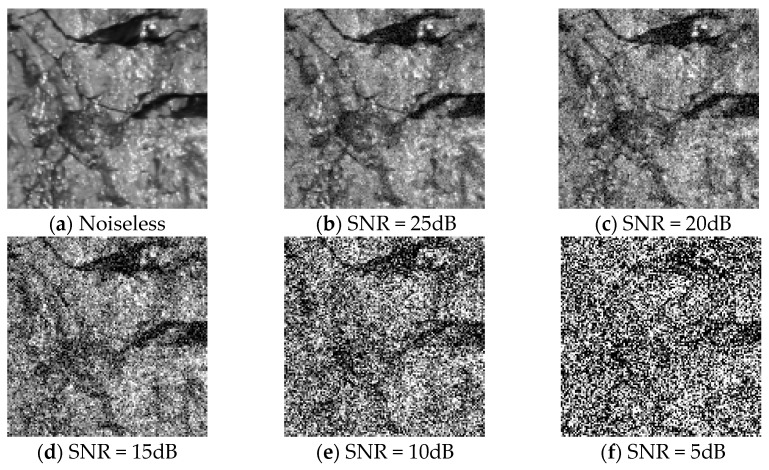
The noiseless image and noise images with different SNR (signal-to-noise ratio) levels. Here, the anthracite gray-scale image is taken as the example. (**a**) The noiseless anthracite gray-scale image; (**b**) The noise anthracite gray-scale image with SNR = 25dB; (**c**) The noise anthracite gray-scale image with SNR = 20dB; (**d**) The noise anthracite gray-scale image with SNR = 15dB; (**e**) The noise anthracite gray-scale image with SNR = 10dB; (**f**) The noise anthracite gray-scale image with SNR = 5dB.

To evaluate the robustness against the noise of the proposed MFFCRR model, we carried out the experiment by adding white Gaussian noise to original coal-rock image samples. Figure 9 shows the original image sample (the noiseless image) and the associated noise images at different signal-to-noise ratio (SNR) levels (5 dB, 10 dB, 15 dB, 20 dB and 25 dB). As seen in Figure 9, the noise images became more and more distorted with the decrease of SNR, which made features extraction more difficult. Table 4 shows the recognition results with macro-average F1 and accuracy metrics at different SNR levels. Note that the noiseless image denotes the original image sample without adding white Gaussian noise in this paper. As shown in Table 4, when less white Gaussian noise is added to the original image, namely higher SNR, the performance of the MFFCRR model degrades a little, although it still meets the present requirements. However, when more and more white Gaussian noise is added (lower SNR), the performance of the MFFCRR model drops dramatically, which does not meet the actual requirements. Hence, experimental results show that the collected coal-rock image should be denoised before applying the MFFCRR model.

### 4.5. Comparison with State-of-the-Art Methods

We compare the performance of the proposed MFFCRR method with state-of-the-art coal-rock recognition methods on the CR dataset, including curvelet transform and compressed sensing method (denoted as CT–CS) [33], CLBP and support vector guided dictionary learning method (denoted as CLBP–SVGDL) [34], and locality-constrained self-taught learning method (denoted as LCSL) [10]. Meanwhile, in order to more comprehensively compare the performance of the proposed MFFCRR model, the TFE sub-model and the DFE sub-model are also used for coal-rock recognition on the CR dataset, respectively (two comparative experiments). In other words, CLBP method (the TFE sub-model using only CLBP) and CNN method (the DFE sub-model using only CNN) are also compared with our method (the MFFCRR model using both CLBP and CNN) at same parameters settings. The comparison results are listed in Table 5.

As shown in Table 5, our proposed MFFCRR method performs much better than state-of-the-art coal-rock recognition methods, both in relation to accuracy and macro-average F1 score, mainly due to the efficient multi-scale feature extraction and fusion techniques we used. Meanwhile, the proposed MFFCRR method based on CLBP and CNN has a better performance than CLBP method or CNN method at same parameters settings, which indicates that multi-scale features fused by the texture features and the deep features are more discriminative than the single texture features or deep features. Therefore, the proposed MFFCRR model is feasible and effective for coal-rock recognition with less data, and has the best recognition accuracy 97.9167% and the best macro-average F1 score 97.3333%, respectively.

## 5. Conclusions and Outlook

In this paper, a MFFCRR model based on CLBP and CNN is proposed to extract and fuse the texture features and deep features of coal-rock images for coal-rock recognition. The TFE sub-model uses CLBP to learn the local texture features, which are used to represent the texture information of coal-rock images, while the DFE sub-model uses CNN to learn the deep features, which are used to provide the macroscopic spatial information of coal-rock images. Then, the texture features and deep features fused together are input to the nearest neighbor classifier with the chi-square distance to realize coal-rock recognition.

The proposed MFFCRR model not only reduces the heavy workload of manual extraction features but also solves the problems of unsatisfactory performance and low robustness in coal-rock recognition methods. Experimental results show the coal-rock recognition accuracy of the proposed MFFCRR method reaches up to 97.9167%, and the MFFCRR model significantly outperforms existing coal-rock recognition methods in terms of the performance metrics (accuracy and macro-average F1 score).

However, coal-rock images are acquired from the coal mine and are easily affected by noise. Therefore, improving the recognition accuracy of the MFFCRR model under low SNR will be the focus of our future work. In the future, we plan to collect more coal-rock images to enrich our dataset, and study more deep models to improve the coal-rock recognition accuracy.

## Figures and Tables

**Table 1:** The experimental results on the MFFCRR (multi-scale feature fusion coal-rock recognition) model using single-scale and multi-scale CLBP (Completed Local Binary Pattern).

P,R	Accuracy (%)	Macro-Average F_1_ (%)
8,1	94.3374	94.1669
16,2	94.6715	94.5173
24,3	94.7917	94.6957
8,1 + 16,2	95.8947	95.0391
8,1 + 24,3	97.9167	97.3333
16,2 + 24,3	95.4169	95.1033
8,1 + 16,2 + 24,3	96.7359	96.3152

**Table 2:** Optimal parameters of the DFE (Deep Feature Extraction) sub-model.

Parameter	Optimization
Batch Size	32
Epochs	100
Dropout	0.5
Learning Rate	0.001

**Table 3:** Impact of two different Activations. ReLU: Rectified Linear Unit; PReLU: Parametric Rectified Linear Unit.

Activation	Accuracy (%)	Macro-Average F_1_ (%)
ReLU	93.3174	92.6117
PReLU	97.9167	97.3333

**Table 4:** Recognition results under white Gaussian noise.

SNR	Accuracy (%)	Macro-Average F_1_ (%)
Noiseless	97.9167	97.3333
25 dB	93.3171	93.1049
20 dB	90.1733	90.0476
15 dB	87.3810	86.8947
10 dB	75.6349	75.1428
5 dB	70.8095	70.2381

**Table 5:** Recognition results comparison with state-of-the-art methods.

Method	Accuracy (%)	Macro-average F_1_ (%)
CT–CS	93.7513	92.9374
CLBP–SVGDL	94.0526	93.9524
LCSL	95.2857	94.7619
CLBP	93.9168	93.1905
CNN	89.4737	89.2281
MFFCRR	97.9167	97.3333
